# New-York Husbands

**Published:** 1860-02

**Authors:** 


### NEW-YORK HUSBANDS.

No observant man who is daily "on 'change," or promenades Wall street will fail of the impression that a large number of our most driving business men drink brandy every day. They seem stuffed, always full, and scarcely a month passes that the morning paper does not make the record of some familiar name: "Died yesterday, suddenly, of disease of the heart." Further on, we learn from the figures that he has passed away in the very prime of life, while he ought to have lived a third of a century longer. A very great deal of this arises from the abandonment of the old-time custom of merchants living in the rear or upper part of the building in which they do business. In fact, "up-town" residences are rapidly working a social ruin. The old-fashioned dinner hour of noon has become the most business portion of the day. Not less than two hours are consumed in going to Union or Madison Squares to dine, and the merchant who does it would soon go to the wall. If he goes without a dinner, the exhaustion consequent would unfit him for the proper performance of his duties. The remedy is "a snack" or "lunch" at an eating-house, and, from various pretenses, porter, ale, beer, wine, or brandy are used now and then, in small quanties at first, steadily increasing in frequency and in amount, until lunch and brandy are indivisible.

Most men of position, principle, and self-respect hesitate long to take brandy at dinner in the presence of wife and children. Many a man would take it abroad, when he would be very far from indulging at home, so that the practice of taking the mid-day meal at an eating-house or a hotel opens an easy door to one of the very worst forms of intemperance, that is, habitual drinking, seldom, if ever, descending to the degradation of actual drunkenness, yet having " brandy aboard" all the time.

One of the finest legal minds in New-York city died not long ago. He dropped suddenly dead. The papers all said of "disease of the heart." His casual acquaintances were not aware of his being an invalid. When they met him on the street he was courteous, frank, and manly. There was an activity in mind and motion, which left the impression of good health and prosperity. But they never saw him early in the morning, nor did his family often, for he seldom appeared at the breakfast table, as he said he had no appetite until late in the day. He rose late, and went directly to his office near Wall street, commencing his day's labor with a large drink of brandy, and it was this which misled those who casually met him; it was on this he lived until noon, when he "took dinner down-town," the almost only meal of the day, made ravenous by the previous potations, and in order to "carry this load," to "settle his dinner," to "aid digestion," he drank brandy largely after dinner, and with this transacted the business of the after part of the day, and in the evening returned to his family, when the double excitement of food and drink made him appear so "full of life," that his own household were misled as to the actual condition of his health, and were not awakened from their delusion until his corpse was one day brought to the door, he having fallen dead in a drinking saloon.

This is no solitary history, no made-up case, for we knew the man and his habits. We have known of other men whose fate was similar. We know men now who are travelling the same road, and who will soon arrive at the same destination!

To every wife in New-York whose husband "dines down-town," this narration should carry with it a lesson and a warning, and however great may be her confidence in her husband, there ought to be some misgivings and some effort to have him dine at home; rather than not do it, the family had a great deal better move down to "the store," and occupy the spacious airy "lofts," as they are called, which, with proper fitting up, would afford a roominess, a cheerfulness, and a pureness of atmosphere equal to the most favored dwellings on the Avenue or Murray Hill. The richest private individual in the world, the Paris Rothschild, lives in the rear of his counting-house. and his western prototype, our olden friend Sayre, the financier, the banker, and the philanthropist, does the same thing, having the wife of his youth literally at his elbow, his parlor and his dining-room opening into his office, and with such propinquity they have grown young in love; age has come without wrinkles, and prosperity without toil, and, like two young turtle-doves in their affections, they are billing and cooing down life's pathway, apparently as happy as the happiest. And who does not know that multitudes in our large cities fail annually in the great aim of their life in consequence of not living on the spot where their business is, in consequence of having houses "up-town" and "out of town," with the additional cost of from three thousand, at least, to ten thousand dollars a year.

If there are insurmountable objections to the plan suggested, there is another, which may be regarded as next best. Let the wife, at least, invite herself down-town to dine with her husband every day, and thus keep away the liquor-bottle; let her cheerfulness, her tidiness, her intelligence, her affection, the brightness of her eyes, and the sweetness of her voice, be the "seasoning" of each meal, the tonic of each repast; let her joyous presence be the whetstone of the appetite, the great exhilerator of the spirits, the great waker-up of those ambitions and energies which are essential to business success.

The dwelling-houses of the business men of New-York are very little more to them than lodging places. It is the custom of some to leave their homes in winter, at least, before the children are up, and return after they are asleep. This is not living; it is more like the unsatisfying life of a man at the galleys. It may be true that this entire consecration to business is not to last many years; that wealth may begin to roll in, and elegant leisure come before "fifty;" but from any hundred in this race for gold, who started at twenty-five, take out the "failures" in money, in character, or in health, not a tithe are left; and of that small number, more than half have had the juices of their affections, their capabilities, and their better natures so eaten out, there is no substance left, there is no capacity for any other enjoyment than that of calculating the cent per cent than that of clutching gold. What a prostitution of the ends and aims of life!

